# Long-term conservation agriculture and best nutrient management improves productivity and profitability coupled with soil properties of a maize–chickpea rotation

**DOI:** 10.1038/s41598-021-89737-9

**Published:** 2021-05-17

**Authors:** Vijay Pooniya, R. R. Zhiipao, Niraj Biswakarma, S. L. Jat, Dinesh Kumar, C. M. Parihar, K. Swarnalakshmi, Achal Lama, A. K. Verma, Debasish Roy, Kajal Das, K. Majumdar, T. Satyanarayana, R. D. Jat, P. C. Ghasal, Hardev Ram, Rajkumar Jat, Amlan Nath

**Affiliations:** 1 ICAR-Indian Agricultural Research Institute (IARI), New Delhi, 110 012 India; 2 ICAR-Indian Institute of Maize Research (IIMR), PAU Campus, Ludhiana, Punjab 141 004 India; 3 ICAR-Indian Agricultural Statistics Research Institute (IASRI), New Delhi, 110 012 India; 4 ICAR-Central Research Institute for Jute and Allied Fibers, Barrackpore, West Bengal 700120 India; 5 African Plant Nutrition Institute (APNI), 43150 Benguérir, Morocco; 6 International Plant Nutrition Institute (IPNI) Centre, New Delhi, 110 012 India; 7 Chaudhary Charan Singh Haryana Agricultural University (CCSHAU), Hisar, Haryana 125004 India; 8 ICAR-Indian Institute of Farming Systems Research (IIFSR), Modipuram, Meerut, 250 110 India; 9 ICAR-National Dairy Research Institute (NDRI), Karnal, Haryana 132001 India; 10 Borlaug Institute for South Asia (BISA), Samastipur, Bihar 848125 India

**Keywords:** Sustainability, Agroecology, Ecosystem services, Restoration ecology

## Abstract

Conservation agriculture (CA)-based practices have been promoted and recouped, as they hold the potential to enhance farm profits besides a consistent improvement in soil properties. A 7 years' field experiment consisting of three crop establishment practices viz., zero-till flatbed (ZTFB), permanent beds (PNB), conventional system (CT) along with the three-nutrient management; nutrient expert-based application (NE), recommended fertilization (RDF), and farmers’ fertilizer practice (FFP), was carried out from 2013 to 2020. The CA-based practices (ZTFB/PNB) produced 13.9–17.6% greater maize grain-equivalent yield (MGEY) compared to the CT, while NE and RDF had 10.7–20% greater MGEY than the FFP. PNB and ZTFB gave 28.8% and 24% additional net returns than CT, while NE and RDF had 22.8% and 17.4% greater returns, respectively over FFP. PNB and ZTFB had 2.3–4.1% (0.0–0.20 m soil layers) lower bulk density than the CT. Furthermore, microbial biomass carbon (MBC) increased by 8–19% (0.0–0.50 m soil layers) in ZTFB/PNB over the CT, and by 7.6–11.0% in NE/RDF over FFP. Hence, CA-based crop establishment coupled with the NE or RDF could enhance the yields, farm profits, soil properties of the maize–chickpea rotation, thereby, could sustain production in the long run.

## Introduction

The importance of conventional rice–wheat cropping system (RWCS) in the Indo-Gangetic Plains (IGPs) in securing food and nutrition has been negated due to the greater water requirement and exacerbating soil fertility status^1^ coupled with the higher production costs and inefficient inputs usages^2,3^. The ever-changing climate and exaggerating soil degradation poses a constant threat to sustainability of the conventional farming practices. The nutritional security of the inhabitants in the region is also impaired due to phasing out of pulses owing to enhanced adoption of a policy backed RWCS. To redress these effects, conservation agriculture (CA)-based practices have been propounded to restore the degrading soil fertility, enhance the resource use efficiency and crops yield improvement^4^.

Maize (*Zea mays* L.), an emerging versatile crop with wider adaptability and photo-insensitivity under the different ecological scenarios, may replace the rice crop in the wet season. It has the potential to address issues such as food and nutritional security^5^, water scarcity, and climate change^4^. Also, there is an increasing demand for maize in the industrial sector with the increased population pressure^6^. Similarly, chickpea (*Cicer arietinum*) is a protein-rich (17–22% of total dry seed mass, and best among all legume proteins) legume, harvested area ~ 13.7 m ha with an annual production of ~ 14.2 m t globally^7^, with the most production centered in India. Maize-based rotations with improved soil and crop management practices have proved a better alternative over RWCS through the realization of better system yields, enhanced soil properties, better utilization and savings of irrigations along with the reduced labor costs^8^. The development of single cross high yielding maize hybrids coupled with the energy-efficient chickpea genotypes have given an ample scope for diversifying the existing cereal-cereal rotations. The inclusion of chickpea in a cereal-based rotation helps in sustaining the soil health and system yields further. It may also help in saving the water over the RWCS.

The conventional crop production model has been deemed unsustainable, as it is less energy efficient, consumes more water with lesser productivity, employs improper inputs usage, and obsolete crop establishment methods^9,10^. Consequently, poor residue management under conventional tillage (CT) practices compelled the farmers either to burn them *in-situ* or feed to the cattle^11,12^. Moreover, intensive tillage not only degrades the soil organic matter due to the enhanced oxidation but also disrupts the organic carbon (SOC), hence impairs the soil properties^13^. The mismanagement and the exacerbating environment through CT practices could be reoriented by adhering to the CA-based practices i.e., no- or minimum tillage, residue retention, and diversifying the crops, thereby, enhancing the soil health and yields^14,15^. Also, adoption of the CA-based crop establishment practices (CEP) in different rotations significantly improves the water use efficiency^16^, system yields^12^, and net returns^10,13,17^. Besides, it improved the soil physical properties^18,19^, built up the SOC^20,21^, and enhanced the soil microbial biomass carbon (MBC)^22^.

Nutrient management in maize–chickpea rotation in the CA-based systems needs to be tailored by accounting for the contribution through the residue retention, atmospheric N-fixation, and residual soil fertility rather than following the usual blanket recommendation. The nutrient expert (NE) based site-specific nutrient management focuses on balanced and crop-need based nutrition^23–25^ and helps in increasing the nutrient use efficiency and provide more profits. This is fundamentally based on the internal nutrient efficiency and gives recommendations based on the QUEFTS (quantitative evaluation of the fertility of tropical soils) model. International Plant Nutrition Institute (IPNI), the NE is user-friendly software that could enhance and sustain the productivity apace with the improved soil health^26–28^. Initially, it was hypothesized that using the NE would enhance the yields and reduce the fertilizer use, and this hypothesis was validated at multi-locations (n = 104) field trials in the southern Indian states by the All India Coordinated Research Project and International Plant Nutrition Institute on maize. The positive results of these validation trials showed that the field-specific fertilizer recommendations based on NE not only increased the crop yields but also optimized the fertilizer application rates^29–31^.

However, the impacts of CA-based CEP coupled with the precise nutrient management using NE in maize–chickpea rotation (MCR) are yet to be evaluated. Therefore, this study was undertaken for 7 years (2013–2020) to assess the impacts of the conservation agriculture and nutrient management practices on the system yields, farm economics, and soil properties in the MCR of north-western India.

## Results

### Weather parameters during the study period

During the cropping seasons (July–April), the highest rainfall of 1368 mm was received in 2013–2014, followed by 1230.2 mm in 2016–2017, while only 600–900 mm was received in 2014–2015, 2015–2016, 2017–2018, 2018–2019 and 2019–2020. Most of the rainfall was received during the three months (July–September), accounting for nearly 80% of the total rainfall. During winters, the common occurrence of the western disturbances in north-western India resulted in very little rainfall. The amount of rainfall received during October–April was least in 2015–2016 (22 mm) and 2017–2018 (39.4), while 100–350 mm was received in the rest of the years (Supplementary Table 1).

### Seven years’ trends and pooled grain and stover yields of maize

**Figure 1:**
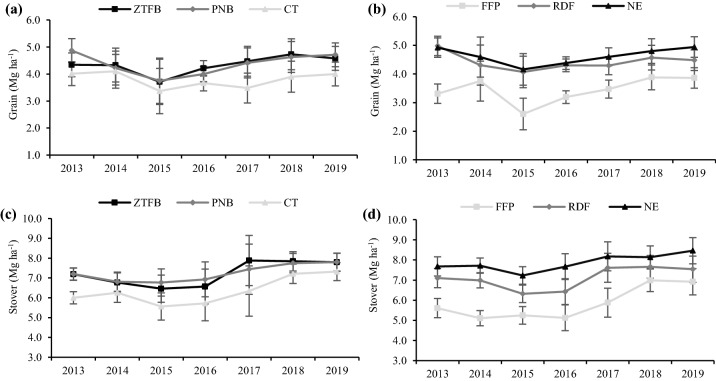
Seven years' grain (**a**,**b**) and stover (**c**,**d**) yields trend of the maize under the CA-based CEP and nutrient management in the maize–chickpea rotation. The vertical bars indicate the LSD at p = 0.05.

The CEP had a significant (*p* < 0.05) effect on the grain and stover yields of maize over the study years. In this study, the initial years showed a reduction in the grain yield, but from the third year onward, the yield increased significantly in the CA over the CT. Grain yield under the zero-tilled flatbed (ZTFB) and permanent beds (PNB) significantly outperformed the CT across the years, except during 2014 and 2015, whereas, the CT had a similar yield to the ZTFB in 2013 (Fig. 1a). Across the years, nutrient expert (NE) and the recommended fertilization (RDF) resulted in a similar yield but being significantly superior to the farmer fertilizer practices (FFP). However, the NE had a significantly higher yield over the RDF during 2019 (Fig. 1b). Based on the pooled data, the PNB and ZTFB had similar grain yield, which was 13.3% and 12.7% higher over the CT, respectively. Similarly, the NE and RDF recorded significantly higher pooled grain yield by 25.7% and 22.3% than the FFP, respectively (Table 1).

**Table 1:** Seven years' mean grain/seed and stover yields (Mg ha^−1^) (± S.D.) and sustainable yield index (SYI) under the CA-based CEP and nutrient management in the maize–chickpea rotation.

Treatment	Maize			Chickpea		
	Grain	Stover	SYI	Seed	Stover	SYI
**CET practices**						
ZTFB	4.34^a#^ ± 0.33	7.22^a^ ± 0.63	0.91^a^	1.41^b^ ± 0.69	4.58^b^ ± 0.56	0.78^b^
PNB	4.37^a^ ± 0.40	7.24^a^ ± 0.43	0.93^a^	1.58^a^ ± 0.70	4.87^a^ ± 0.57	0.89^a^
CT	3.79^b^ ± 0.29	6.34^b^ ± 0.69	0.79^b^	1.24^c^ ± 0.48	4.08^c^ ± 0.44	0.68^c^
**Nutrient management**						
FFP	3.44^c^ ± 0.46	5.84^c^ ± 0.81	0.61^c^	1.31^b^ ± 0.63	4.06^c^ ± 0.60	0.82^c^
RDF	4.43^a^ ± 0.30	7.09^b^ ± 0.55	0.82^a^	1.43^a^ ± 0.65	4.43^b^ ± 0.56	0.90^a^
NE	4.63^a^ ± 0.29	7.87^a^ ± 0.41	0.86^a^	1.49^a^ ± 0.57	5.03^a^ ± 0.42	0.94^a^

^#^Means followed by a similar uppercase letter within a column is not significantly different (p < 0.05) according to Tukey’s HSD test.

In all the years, the ZTFB and the PNB had similar stover yields, but being significantly higher than the CT (Fig. 1c), and had 12.4% and 12.2% higher pooled yield over the CT, respectively. The stover yield under the NE and RDF, was significantly higher over the FFP across the years, whereas the NE also had a significantly better stover yield than the RDF, except during 2017 and 2018 (Fig. 1d). The 7 years' average showed that the NE and RDF produced 25.8% and 17.6% higher stover yields than the FFP, respectively (Table 1).

### Seven years’ trends and pooled seed and stover yields of chickpea

**Figure 2:**
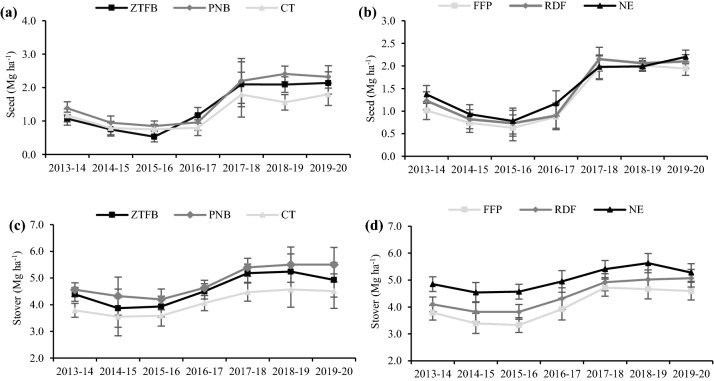
Seven years' seed (**a**,**b**) and stover (**c**,**d**) yields trend of the chickpea under the CA-based CEP and nutrient management in the maize–chickpea rotation. The vertical bars indicate the LSD at p = 0.05.

In all the years, the PNB produced maximum seed yield, except in 2016–2017 (Fig. 2a), followed by the ZTFB. The NE was the best, except during 2017–2018 and 2018–2019, with no difference among the FFP, RDF, and NE in 2014–2015, 2015–2016, 2017–2018, and 2018–2019 (Fig. 2b). The PNB had 10.8% and 21.5% greater pooled seed yield than the ZTFB and CT, respectively (Table 1).

The PNB and ZTFB had similar chickpea stover yield, being significantly (*p* < 0.05) greater than the CT, but in 2014–2015, 2015–2016, and 2019–2020, the ZTFB was at par to the CT (Fig. 2c). In contrast, the NE recorded a significantly greater stover yield than the RDF and FFP but was at par with the RDF in 2019–2020 (Fig. 2d). The PNB had registered maximum stover yield, which was significantly higher over the ZTFB and CT. However, the NE had 11.9% and 19.3% greater stover yield over the RDF and FFP, respectively (Table 1).

### System productivity as maize grain equivalent yield (MGEY)

**Figure 3:**
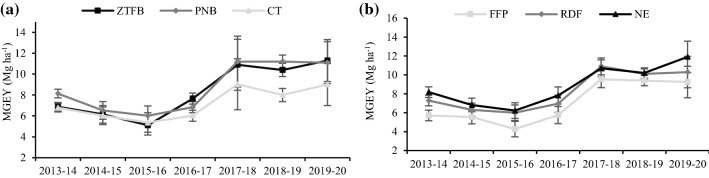
Seven years' trend in the MGEY (**a**,**b**) under the CA-based CET and nutrient management in the maize–chickpea rotation. The vertical bars indicate the LSD at p = 0.05.

The PNB had significantly (*p* < 0.05) greater MGEY over the ZTFB (2013–2014, 2018–2019) and CT, but in 2014–2015 and 2015–2016; no significant difference among CEP practices was observed. Thereafter, in 2016–2017, the ZTFB had significantly higher MGEY (10.7–21.4%) compared to the PNB and CT; while in 2017–2018 and 2019–2020, the ZTFB and PNB had similar yields (Fig. 3a). In nutrient management, the NE and RDF were comparable for the MGEY across the years but significantly greater than the FFP. However, in 2019–2020, the RDF and FFP had similar MGEY. Averaged across the 7 years, the ZTFB and PNB produced 13.9% and 17.6% greater MGEY than the CT, however, the NE and RDF gave 10.7% and 20% greater MGEY than the FFP, respectively (Fig. 3b).

### Interaction effect (CEP × nutrient) on the MGEY

The CEP and nutrient management had a significant (*p* < 0.05) interaction effect on the MGEY across the years. In 2013–2014, the PNB–NE had maximum MGEY, and at par with the PNB–RDF and CT–RDF. Similarly, in 2014–2015, the PNB–NE produced the highest MGEY but did not differ from the PNB–RDF, ZTFB–NE, ZT–RDF, and CT–NE. In 2015–2016, the PNB–NE had a similar yield to all the treatment combinations, except CEP with FFP. In contrast, the ZTFB–NE had the greatest MGEY during 2016–2017, 2017–2018, and 2019–2020. However, it was at par with the RDF and NE, irrespective of the CEP during 2016–2017, 2017–2018, and 2019–2020. Whereas in 2018–2019, the PNB–NE exhibited the highest MGEY, and significantly greater than the ZTFB–FFP and CT (Table 2). The results of the current study also suggested that the NE and RDF either with the PNB or ZTFB tended to have relatively more MGEY than the with CT.

**Table 2:** Interaction effect of the CA-based CEP and nutrient management on the system productivity (± S.D.) in terms of MGEY during the 7 years of the study in the maize–chickpea rotation.

Treatment	Maize grain equivalent yield (MGEY, Mg ha^−1^)						
	2013–2014	2014–2015	2015–2016	2016–2017	2017–2018	2018–2019	2019–2020
ZTFB–FFP	6.35^cde^ ± 0.31	5.39^c^ ± 0.12	3.79^d^ ± 0.66	6.33^bcde^ ± 1.18	9.18^cd^ ± 1.23	9.5^b^ ± 0.52	8.51^c^ ± 0.45
ZTFB–RDF	6.76^cd^ ± 0.15	6.26^abc^ ± 0.69	5.48^abc^ ± 0.36	7.84^ab^ ± 0.80	11.5^ab^ ± 1.54	10.9^a^ ± 0.40	11.2^bc^ ± 0.88
ZTFB–NE	7.54^bc^ ± 0.37	6.82^ab^ ± 1.10	6.08^ab^ ± 0.60	8.83^a^ ± 0.85	12.3^a^ ± 0.69	11.1^a^ ± 0.41	14.4^a^ ± 4.59
PNB–FFP	5.82^de^ ± 0.07	5.49^bc^ ± 0.30	4.56^bcd^ ± 0.71	5.79^de^ ± 1.02	10.4^bc^ ± 1.21	10.9^a^ ± 0.77	10.1^bc^ ± 0.84
PNB–RDF	9.07^ab^ ± 0.12	6.63^abc^ ± 0.67	6.72^a^ ± 0.87	6.93^bcd^ ± 1.67	11.8^ab^ ± 1.26	11.3^a^ ± 0.43	11.1^bc^ ± 0.51
PNB–NE	9.54^a^ ± 0.77	7.48^a^ ± 0.34	6.79^a^ ± 0.70	7.81^abc^ ± 0.91	11.5^ab^ ± 1.27	11.6^a^ ± 0.72	12.3^ab^ ± 0.40
CT–FFP	4.96^e^ ± 0.96	5.77^bc^ ± 1.64	4.48^cd^ ± 0.84	5.13^e^ ± 0.61	9.12^cd^ ± 1.43	7.87^c^ ± 0.29	9.16^c^ ± 0.5
CT–RDF	7.93^abc^ ± 0.75	6.01^bc^ ± 0.13	5.81^abc^ ± 1.07	6.11^cde^ ± 0.70	9.64^cd^ ± 1.23	8.27^c^ ± 0.09	8.73^c^ ± 1.07
CT–NE	7.48^bc^ ± 0.19	6.29^abc^ ± 0.29	5.82^abc^ ± 0.68	6.84^bcde^ ± 0.49	8.34^d^ ± 0.81	7.89^c^ ± 0.37	9.16^c^ ± 0.49

Means followed by a similar uppercase letter within a column is not significantly different (p < 0.05) according to Tukey’s HSD test.

### Farm economics

The 7 years' mean data indicated that the CT (US$ 639.9 ha^−1^) was the most expensive CEP, which was 15.3% and 16.9% costlier than the PNB (US$ 541.7 ha^−1^) and ZTFB (US$ 531.6 ha^−1^), respectively. Likewise, the RDF (US$ 582.7 ha^−1^) accounted for the highest cultivation cost, closely followed by the NE (US$ 576.7 ha^−1^), being 5% and 4.1% higher over the FFP (US$ 553.3 ha^−1^), respectively. In all the years, the PNB had the highest net returns, whereas, in 2016–17, the ZTFB accounted for the greater returns. However, the ZTFB and PNB did not differ for the system net returns, except during 2013–2014, 2016–2017, and 2018–2019.

The PNB (US$ 1671.1 ha^−1^) and ZTFB (US$ 1565.9 ha^−1^) had generated 28.8% and 24% higher net returns than the CT (US$ 1189.8 ha^−1^), respectively. Similarly, the NE (US$ 1635.9 ha^−1^) and RDF (US$ 1528.4 ha^−1^) had 22.8% and 17.4% greater net returns than the FFP (US$ 1262.9 ha^−1^) (Table 3), respectively.

**Table 3:** Economics of the maize–chickpea rotation under the CA-based CEP and nutrient management during the 7 years of the study.

Treatment	Variable production costs (US$ ha^−1^ year^−1^)							System net returns over production costs (US$ ha^−1^ year^−1^)						
	2013–2014	2014–2015	2015–2016	2016–2017	2017–2018	2018–2019	2019–2020	2013–2014	2014–2015	2015–2016	2016–2017	2017–2018	2018–2019	2019–2020
**CET practices**														
ZTFB	470^c^	513^c^	517^c^	508^c^	580^c^	532^c^	601^c^	1104^b^	945^ab^	754^a^	1672^a^	2086^a^	2204^b^	2196^a^
PNB	476^b^	520^b^	525^b^	516^b^	585^b^	548^b^	622^b^	1362^a^	1025^a^	865^a^	1513^b^	2126^a^	2378^a^	2429^a^
CT	554^a^	614^a^	630^a^	621^a^	694^a^	642^a^	724^a^	979^c^	805^b^	575^b^	1327^c^	1499^b^	1551^c^	1593^b^
**Nutrient management**														
FFP	475^c^	525^c^	535^c^	530^c^	609^c^	563^c^	636^c^	828b	775^b^	479^b^	1263^c^	1707^b^	1910^b^	1878^c^
RDF	516^a^	565^a^	573^a^	560^a^	626^a^	581^a^	658^a^	1269a	926^ab^	804^a^	1527^b^	2018^a^	2100^a^	2055^b^
NE	508^b^	556^b^	564^b^	554^b^	624^a^	578^a^	653^a^	1349a	1074^a^	911^a^	1723^a^	1986^a^	2123^a^	2285^a^

Means followed by a similar uppercase letter within a column is not significantly different (*p* < 0.05) according to Tukey’s HSD test. Price of one US $ = 62.9 INR2013–2014; 60.9 INR2014–2015; 64.2 INR2015–2016; 68 INR2016–2017; 64 INR2017–2018; 74 INR2018–2019; 70 INR2019–2020.

### Sustainable yield index (SYI)

Among the CEP in maize, the PNB had the greater SYI, but being at par to the ZTFB, which was 15% and 13.2% greater than the CT. Further, SYI in maize was the highest under the NE, similar with RDF, being 29.1% and 25.6% greater than the FFP. In the case of chickpea, the SYI was highest under the PNB, which was 12.4% and 23.6% higher than the ZTFB and CT, respectively. The SYI in the NE and RDF were at par, being 8.9–12.8% greater than the FFP in chickpea (Table 1).

### System water productivity (SWP)

**Figure 4:**
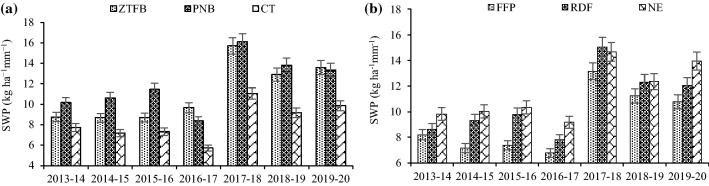
Seven years' trends of SWP (**a**,**b**) in the CA-based CEP and nutrient management under the maize–chickpea rotation. The vertical bars indicate the LSD at p = 0.05.

During the first 3 years of the study, the PNB (10.1–11.5 kg ha-mm^−1^) led to the highest SWP, which was significantly higher than the ZTFB (8.7 kg ha-mm^−1^) and CT (7.2–7.7 kg ha-mm^−1^), respectively. Whereas, fourth year onward, the PNB (8.4–16.1 kg ha-mm^−1^) had a similar SWP to the ZTFB (9.7–15.7 kg ha-mm^−1^), but significantly higher than the CT (5.7–11.1 kg ha-mm^−1^) (Fig. 4a). Among nutrient management, the NE and RDF had similar SWP, except during 2013–2014, 2016–2017, and 2019–2020 however, it was 20% and 14% (7 years' mean) greater than the FFP (Fig. 4b).

### Soil bulk density (*ρ*b), organic carbon (SOC) and microbial biomass carbon (MBC)

**Figure 5:**
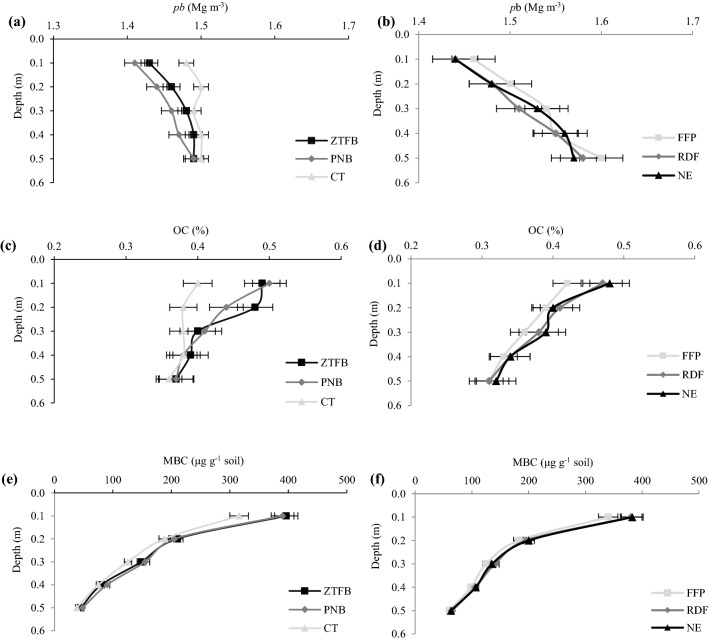
Soil *p*b (**a**,**b**), OC (**c**,**d**) and MBC (**e**,**f**) under the CA-based CEP and nutrient management after 7th season maize in the maize–chickpea rotation. The horizontal bars indicate the LSD at p = 0.05.

After harvest of the seventh season maize, soil samples were collected from the 0.0–0.50 m soil depth (Fig. 5a). Among the CEP, the PNB and ZTFB had significantly lower *ρ*b than the CT up to the 0.20 m soil depth. The decrement was to the tune of 2.3–4.1% over the CT, though there was no difference in the ZTFB and PNB. In contrast, beyond the 0.20 m soil depth, *ρ*b did not differ significantly among the CEP (Fig. 5a). Across the soil profile, nutrient management practices were at par for *ρ*b (Fig. 5b). The CEP had a significant (*p* < 0.05) impact on the SOC up to the 0.20 m soil depth, the ZTFB and PNB were at par, but greater than the CT at 0.0–0.20 m depth. However, there was no difference in the 0.20–0.50 m soil section (Fig. 5c). The NE and RDF had similar values for the SOC, but significantly greater than the FFP at 0.0–0.10 m depth, however, the SOC did not differ among the nutrient management beyond the 0.10 m soil depth (Fig. 5d).

At the silking stage of the seventh season maize, the ZTFB and PNB had similar values for the MBC, but significantly greater by 8.3–20.3% than the CT at 0.0–0.30 m soil depth (Fig. 5e). Likewise, the NE and RDF had similar MBC, but significantly greater than the FFP at 0.0–0.10 m depth. However, beyond the 0.10 m soil depth, these practices did not show a significant influence on the MBC (Fig. 5f).

**Figure 6:**
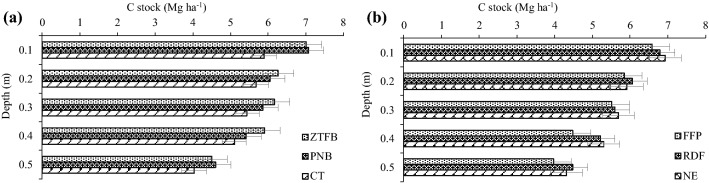
Effect of tillage (**a**) and nutrient management (**b**) on SOC stocks (equivalent soil mass basis) of maize–chickpea rotation. The vertical bars indicate LSD at p = 0.05.

The ZTFB and PNB had 18.8–19.8% higher SOC stock (Mg ha^−1^) at 0.10 m soil layer, but in the at 0.10–0.50 m soil depth, CEP did not have a significant impact on it, though relatively greater values across the depths were recorded in the ZTFB/PNB than the CT. Nevertheless, the total SOC stock up to the 0.50 m soil depth was 10.9–14.2% greater with ZTFB/PNB than the CT (Fig. 6a). The NE had the highest SOC stock across the soil layers (0.0–0.50 m), which was similar to the RDF, but 6.7% greater than the FFP (Fig. 6b).

## Discussion

Diversifying the existing dominant rice–wheat cropping system (RWCS) towards maize-based, particularly maize followed by legumes under the CA-based CEP (ZTFB/PNB) along with the balanced nutrition, could enhance and stabilize yields and the farm profits^12,32^ besides the improved soil properties in long-run^33^. RWCS in the IGPs of South Asia is facing challenges of exaggerating decline in the groundwater table and the input use efficiencies^34^. Henceforth, maize–chickpea rotation (MCR) has the potential to combat the twin challenges of the declining groundwater by 30–40 cm year^−1^^35^, and the import of pulses for nutritional security. In this study, the CA-based CEP had greater maize (12–13%) and chickpea (12–21%) yields across the years over the CT. It could be associated with the commendatory soil temperature/moisture conditions^36^, improved soil properties^13,37^, better water and nutrient uses^18^ besides, amalgamating the effects of the residue retention^38^. Further, these practices also help in the better retention and infiltration of the water and favour better growing conditions that may have resulted in greater crop yields.

Despite the wide variability in the precipitation (excess/deficit ranis) during the study, the PNB/ZTFB recorded ~ 13.9–17.6% (7 years mean) greater MGEY than the CT. The MGEY had strong relationship with the SOC stock under the CA-based CEP (r^2^ = 0.87; *p* < 0.05) and the nutrient management (r^2^ = 0.94; *p* < 0.05). So, due to the improved levels of SOM/SOC stocks and other soil properties, reinforcing the previous findings in the cereal-based rotations^9,10,19^. In this study, relatively higher mean yields were exhibited in the NE, however being comparable to the RDF. The yield differences in the NE and RDF could be due to the variation in the fertilizer rates, besides the NE entailed applications of the balanced and location-specific, which is fundamentally based on the nutrient carrying capacity, thereby it may have enhanced the internal nutrient efficiency^12,19,39^. Possibly, optimal nutrition would have led to the better partitioning of the photosynthates, thereby, more vigorous plant growth with the stiffer rooting and greater resistance against the abiotic stresses^40^. Residual fertility in the NE and RDF was outlined with the higher chickpea yield over the FFP. Results of multi-location trials in South Asia had shown that the NE gave greater yields under the CA than the CT system^24,30,41^.

The feasibility of any technology/management practice could be assessed ultimately through farm economics. In this study, the CT incurred a higher cultivation cost by US$ 84–123 ha^−1^ compared to the ZTFB/PNB. This higher cost in CT was mainly attributed to the additional tillage^10^, apart from the higher labour cost needed for extra intercultural operations. On the contrary, the increment in the returns under the ZTFB/PNB was to the tune of US$ 380–481 ha^−1^ over the CT. The CT had the higher farm cultivation cost with the lower MGEY, which in turn reflected in the lower net returns^10,42,43^. Greater net returns under the NE could be due to a balanced and crop need based fertilizer application^24,25^ resulting in more yields, and the returns. The comparative field studies (n = 82) of the NE with the state recommendation and FFP in Southern India, reasoned out that, farmers’ risk could well be reduced, when the NE was adopted, as it directs and provides a proper and balanced rate of fertilizers^30^. Hence, optimized nutrient uses apace with the higher yields and profitability under the maize and maize-based rotations.

Concerning SWP, the ZTFB and PNB had 25.6% and 30.9% greater values than the CT, which could be ascribed to the better soil moisture regimes due to the surface residue retention coupled with a higher yield gains^37,44^. Furthermore, higher SOC stock in the CA-based CEP enhanced the moisture retention and opportune time for the water movement in soil^45,46^, hence it facilitates a greater water and nutrient acquisition and ultimately SWP. The major impacts of the CA-based CEP are conspicuous through higher SOM, especially in the topsoil layers apace with the better soil structural stability and biological diversity in contrast to the CT systems^47^. After 7 years, the ZTFB and PNB with residues improved the OC stock at 0.0–0.10 m depth by 18.8–19.8%, and total OC stock by 10.9–14.2% than the CT. This would be associated with better physical protection of particulate organic matter; greater residues remain on the soil surface coupled with a lesser turnover of macro-aggregates as well as minimal contact between residue and soil^48^. Extensive tillage reduces SOC, as it breaks open the previously protected organic matter leading to increased microbial decay^49,50^, as observed under the CT system. The implications of the higher SOC under the NE/RDF could well be attributed to the proper growth and development of the crop, hence greater above/below-ground biomass production, and eventually the increases in organic matter over the FFP.

The effect of the CA-based CEP on the bulk density (*ρ*b) had shown contradictory results, with some studies reported higher *ρ*b^51,52^, on the contrary, some had reported lower *ρ*b^53^ or no changes^54,55^ relative to the CT system. Nevertheless, in our study, the ZTFB/PNB with residues brought down soil *ρ*b by 2.3–4.1% in the top 0.0–0.20 m soil layer, while the nutrient management did not differ for soil *ρ*b. The lower *ρ*b associated with the residues retention leads to greater soil faunal activities^51,53^, thereby, resulting in better soil aggregation and porosity. In contrast, the increased *ρ*b under the CT is due to the compaction particularly in the plough soil layer^13,19^. The MBC depicts the nutrient cycling ability of soil under different management practices^56,57^ in concurs with the organic matter content. The CA-practices coupled with the NE/RDF favours build-up of the SOC through, rhizo-deposition of root stubbles^39,58^, which certainly increased the MBC^59^ and crop yields^60,61^. Besides, the greater organic matter would expedite the soil MBC and other biological activities^62^. This 7 years’ study indicates synergy between the CA-based CEP and NE or RDF through improvements in yields, MGEY, farm returns, and SWP apart from the soil properties. Also, this is well supported by a greater SYI, hence could be propounded for its adoption at present and in posterity.

## Conclusions

The CA-based CEP (ZTFB/PNB) apace with the enhanced resource use-efficiency should be a norm, not the exception, as clearly outlined in our 7 years' experiment, wherein, the ZTFB/PNB with the NE or RDF excelled for the system yields, net returns, SWP, *ρ*b, SOC and SYI. The 7 years' mean showed that the MGEY under the ZTFB and PNB increased by 13.9–17.6%, respectively compared to the CT, however, the NE and RDF registered 10.7**–**20% greater MGEY than the FFP. Furthermore, the CA-based CEP along with the NE or RDF gave an additional net return of US$ 376–481 ha^−1^ year^−1^ and US$ 265–373 ha^−1^ year^−1^ (7 years' av.) than the CT**–**FFP, respectively. Also, these practices significantly improved the SOC stock (18.8–19.8%) and MBC (8–19%) with lower *ρ*b (2.3–4.1%) in the topsoil layers. The improved soil properties coupled with the greater yields was well substantiated with the simultaneous improvement in SWP under the ZTFB (7.5%) and PNB (30.8%). The greater SYI also signified the superiority and sustainability of the rotation in the long-run. Thus, the ZTFB/PNB with the NE or RDF in the maize–chickpea rotation can be well adopted in the semi-arid Indian ecologies to realize its several benefits under the changing climate.

## Materials and methods

### Experimental site

A field experiment on the maize–chickpea rotation (MCR) was established during the rainy season of 2013 at the research field of the ICAR-Indian Agricultural Research Institute (28° 38′ N; 77° 09′ E; 228.6 m MSL), New Delhi, India. Before the establishment of this study, a uniformity field trial on wheat was conducted during the winters of 2012–2013 (November–April). The climate of the region is semi-arid and experiences the dry-hot summers and cold winters. During the growing season of maize (July–October) and chickpea (October–April), the maximum rainfall of 1368 mm was received in 2013–2014, while the lowest of only 604.6 mm in 2017–2018. On average, ~ 80% of the total rainfall was received from the southwest monsoon (July–September), and the average relative humidity across the years ranged between 69 and 87%. The minimum and maximum temperatures during the cropping period ranged from 5 to 28 °C and 18–38 °C, respectively (Supplementary Table 1). The Typic Haplustept sandy loam soil of the experimental site had 7.31 pH, 0.40% Walkley–Black carbon^63^, 159.9 kg ha^−1^ alkaline KMnO_4_ oxidizable-N^64^, 15.6 kg ha^−1^ NaHCO_3_ extractable-P^65^, and 161.3 kg ha^−1^ NH_4_OAc extractable-K^66^.

### Experimental treatment details

**Figure 7:**
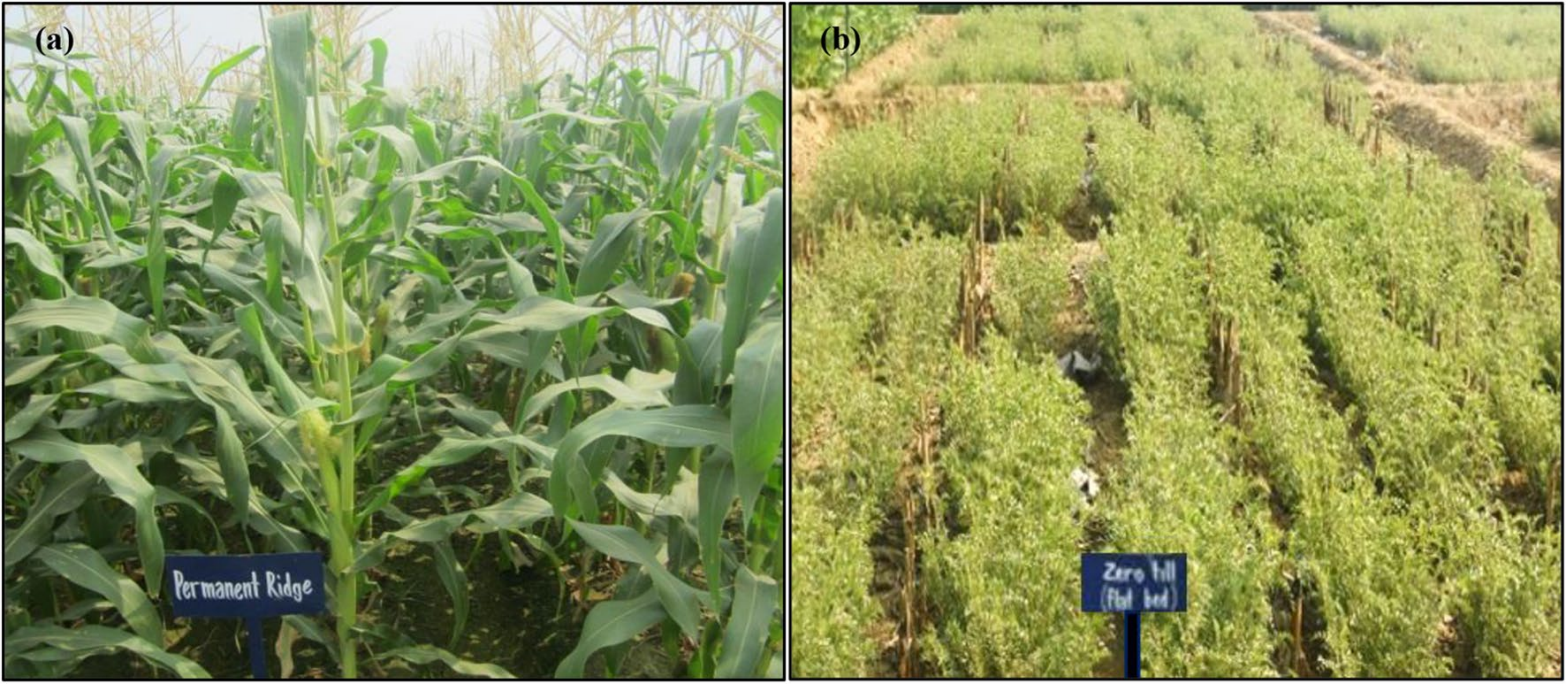
Maize–chickpea rotation under the CA and nutrient management – PNB maize (**a**) and ZTFB chickpea (**b**).

This field experiment consisted of the combinations of three CEP; zero-till flatbed (ZTFB), permanent beds (PNB) and conventional tillage (CT) in main-plots, and three nutrient management practices; farmers’ fertilizer practices (FFP), recommended fertilization (RDF), and nutrient expert assisted-site specific nutrient management (NE) in sub-plots. A split-plot design was employed for the fixed plot experiment with three replicates during the entire study period (Table 4). Before the start of the experiment, the field was deeply ploughed using a chisel plough (~ 0.30–0.45 m) and laser leveled. The CT plots involved one ploughing (~ 0.25–0.30 m), followed by harrowing/rotavator (~ 0.15–0.20 m) and then leveling, while for the ZTFB, no ploughing was accomplished. Initially, the PNB was prepared using the ridge maker (0.67 m), and subsequently the disc coulter once in year before the maize sowing for the reshaping of the beds during each following crop season. The gross plot size under each CEP practice was 20 m × 8.5 m. Maize at maturity was harvested from 0.40 m height and standing residues were retained in the field under the ZTFB/PNB plots. Similarly, the chickpea residues ~ 2.5 Mg ha^−1^ (root stubbles along with the above-ground stover) on a dry weight basis were retained in the plots (Fig. 7).

**Table 4:** Description of the crop establishment practices (CEP) and nutrient management adopted in the maize–chickpea rotation during the 7 years of experimentation.

S. no.	Treatment	Treatment notations	Residue/nutrient management
1	Zero till flat bed	ZTFB	Chickpea (root stubbles + above ground) residues ~ 2.5 Mg ha^−1^ and maize stubbles of ~ 0.40 m height from ground were retained
2	Permanent beds (0.37 m beds and 0.30 m furrows)	PNB	Chickpea (root stubbles + above ground) residues ~ 2.5 Mg ha^−1^ and maize stubbles of ~ 0.40 m height from ground were retained
3	Conventional tillage	CT	Full residue removed and conventional tillage practices followed
4	Farmer’s fertilizers practice	FFP	Maize: 110:13:2.0 kg NPK ha^–1^; chickpea: 18:20:0 kg NPK ha^–1^
5	Recommended dose of fertilizers	RDF	Maize: 150:26.2:50 kg NPK ha^–1^; chickpea: 20:26.2:16.6 kg NPK ha^–1^
6	Nutrient expert assisted: site-specific nutrient management (7 years' mean)	NE	Maize: 130:19.5:55 kg NPK ha^–1^; chickpea: 20:26.2:16.6 kg NPK ha^–1^

### Crop management practices

A quality protein maize hybrid 'HQPM1' was used for the experiment, which was later replaced by the hybrid 'PMH1' in 2017. In each cropping season, the maize seeds were dibbled manually in the rows at a spacing of 0.67 × 0.20 m during the first week of July. Similarly, sowing of the chickpea genotype 'Pusa 372' was done at the end of October in each year i.e., after the maize harvest. Under the RDF practice, a full dose of phosphorous (P) by di-ammonium phosphate (DAP) and potassium (K) using muriate of potash along with the nitrogen (N) was applied as basal to both the maize and chickpea crops, after that two equal N splits to maize were applied at the knee-high and flowering stages. The fertilizer doses for FFP were based on the Participatory Rural Appraisal (PRA) i.e., how most of the farmers’ in the region follow fertilization to the crops. While, for NE treatment, fertilizer rates/splits were followed as per the NE software, available at the http://www.ipni.net. Fertilizers were placed basally in the topsoil layer, while the required N was top-dressed in bands near to the rows (side dressing). Since there is no NE for chickpea, hence, fertilizers were applied as per the RDF in the NE sub-plots. The non-selective herbicide glyphosate at 1 kg ha^−1^ was sprayed about a week before the sowing of the crops both in the ZTFB/PNB plots. A pre-emergence (2 days after sowing, DAS) atrazine (maize) and pendimethalin (chickpea) was sprayed; manual weeding’s were done in the CT. Crop protection practices such as pest and disease management were followed as per the requirement of both crops. Irrigation water was applied considering the rainfall pattern coupled with critical crop stages, in which maize crop received three to six irrigations, while for chickpea it was one to two per crop season.

### Soil sampling and processing

Five soil samples were collected from the 0.0–0.30 m depth (June 2013) for initial analysis, and after harvest of 7th season maize from different soil sections i.e., 0.0–0.10, 0.10–0.20, 0.20–0.30, 0.30–0.40 and 0.40–0.50 m up to 0.50 m depth using the core. The samples were shade dried and ground gently using the wooden pestle and mortar, sieved in a 2 mm sieve, and stored in the air-tight polythene for further analysis of soil properties. For soil MBC analysis, the moist soil samples were collected using the tube auger from 0.0–0.50 m depth at 0.10 m intervals at the silking stage of maize. Finely sieved soil samples were stored at 5 °C (18–24 h) for MBC analysis.

### Soil organic carbon

The soil organic carbon (SOC) was determined by the chromic acid wet oxidation method^63^. The finely ground and sieved soil from the different depths (0.0–0.10, 0.10–0.20, 0.20–0.30, 0.30–0.40, and 0.40–0.50 m) was used for its determination. The SOC in 0.10 m intervals up to 0.50 m soil depth was computed by using Eq. (1)^67^

1 $$ {\text{SOC stock }}\left( {{\text{Mg ha}}^{{ - {1}}} } \right) = {\text{SOC }}\left( \% \right)/{1}00 \times {\text{d }}\left( {\text{m}} \right) \times \rho {\text{b }}\left( {{\text{Mg m}}^{{ - {3}}} } \right) \times {\text{a }}\left( {{\text{ha}}} \right) $$

where, d denotes the soil depth (m) and a is the area (ha).

The SOC stocks on an equivalent soil depth basis were estimated using the following equation:

2 $$ {\text{SOC stock }}\left( {{\text{Mg ha}}^{{ - {1}}} } \right) = {\text{total SOC content }}\left( {{\text{g kg}}^{{ - {1}}} } \right) \times \rho {\text{b }}\left( {{\text{Mg m}}^{{ - {3}}} } \right) \times {\text{depth }}\left( {\text{m}} \right) \times {1}0 $$

To prevent bias due to variation in soil *ρ*b (Mg m^−3^) and take into full account the effect of soil mass on SOC stock^68^. Thus, we have calculated total SOC stocks on an equivalent soil mass (ESM) using formula^69^

3 $$ {\text{Error term }}\left( {{\text{ET}}} \right) = {\text{total SOC concentration }}\left( {{\text{g kg}}^{{ - {1}}} } \right) \times \left( {{\text{M}}_{{{\text{soil}}}} - {\text{ESM}}} \right) \times {1}0 $$

where, M_soil_ is the soil mass and ESM is the equivalent soil mass

4 $$ {\text{ESM }}\left( {{\text{Mg m}}^{{ - {2}}} } \right) = {\text{initial soil}}\;\rho {\text{b }}\left( {{\text{Mg m}}^{{ - {3}}} } \right) \times {\text{depth }}\left( {\text{m}} \right) $$

5 $$ {\text{Msoil }}\left( {{\text{Mg m}}^{{ - {2}}} } \right) = {\text{soil }}\rho {\text{b }}\left( {{\text{Mg m}}^{{ - {3}}} } \right) \times {\text{depth }}\left( {\text{m}} \right) $$

6 $$ {\text{Total SOC on ESM basis }}\left( {{\text{Mg ha}}^{{ - {1}}} } \right) = {\text{total SOC on soil depth basis}} - {\text{ET}} $$

### Soil bulk density

Soil bulk density (*ρ*b) was measured from the different soil sections to a profile depth of 0.50 m, and five samples were collected randomly from each plot using the core sampler. The collected samples were then dried at 105 °C for 48–72 h until a constant weight reached, and then the soil *ρ*b (Mg m^−3^) was computed using equation^4,7^:

7 $$ \rho {\text{b}} = {\text{M}}s{\text{/V}}c $$

where, Ms indicates the dry weight of the sample, and V*c* is the volume of the core.

### Soil microbial biomass carbon (MBC)

The fumigation extraction^70^ method was employed to analyse the MBC in soil samples. The pre-weighed soil samples were taken in a closed capped amber colour bottle and fumigated with the ethanol-free chloroform. The un-fumigated samples were also taken in a transparent capped bottle and maintained. Both the fumigated and non-fumigated samples were incubated in the dark for 24 h, after which fumigated samples were evaporated by opening the cap and exposing it to the sun for ~ 20–25 min. and later in a hot air oven at 50 °C for ~ 20 min. The processed samples were added 0.5 M K_2_SO_4_ (soil:extractant 1:4) and kept on a mechanical shaker for 30 min. and soil suspension was filtered using the Whatman No. 42 filter paper. The carbon content was determined through dichromate digestion superseded by back titration with 0.05 N ferrous ammonium sulphate, then the MBC content was computed using equation:

8 $$ {\text{MBC }}(\upmu {\text{g C g}}^{{ - {1}}} {\text{soil}}) = {\text{EC}} \times {2}.{64} $$

where, EC = (C in fumigated soil – C in un-fumigated soil).

### Yield measurements

During the 7 years of the experimentation, the crop yields were estimated from the net plot area, leaving the border rows in both the crops. The border plot area was harvested first, and then the net plot area for recording the grain/seed yields. The harvested maize cobs were sundried for 45–50 days and the stover for about a month in the open field conditions and then threshed by a mechanical thresher. The chickpea was harvested manually, and the harvested produce was sundried, then threshed using a tractor-drawn pull man thresher. For grain yield, the moisture content was adjusted to ~ 12% in both crops. The stover/stalk yields were obtained by subtracting the grain/seed yields from their respective total biomass yield. To estimate the system productivity of MCR, chickpea seed yield was converted into the maize grain equivalent yield (MGEY) as given in Eq. (9)^10,61^.

9 $$ {\text{MGEY }}\left( {{\text{Mg ha}}^{{ - {1}}} } \right) = {\text{Y}}_{{\text{m}}} + \left\{ {\left( {{\text{Y}}_{{\text{c}}} \times {\text{P}}_{{\text{c}}} } \right) \div {\text{P}}_{{\text{m}}} } \right\} $$

where, MGEY = maize grain equivalent yield (Mg ha^−1^), Y_m_ = maize grain yield (Mg ha^−1^), Y_c_ = chickpea seed yield (Mg ha^−1^), P_m_ = price of maize grain (US$Mg^−1^) and P_c_ = price of chickpea seed (US$Mg^−1^).

### Farm economics

The total production cost was computed based on the variable costs for each treatment. The cost included human labour employed for different field operations, rental land value, use of machinery viz. tractor, plough, planter, thresher, etc., fertilizers, seed, pesticides, other plant protection chemicals, irrigations, and harvesting. The gross returns included the market value of both grain/seed and stover/stalk, wherein the value of grain/seed was as per the minimum support price set by the Government of India during the respective seasons. The net returns were computed using the formula: net returns (US$ ha^−1^) = [gross returns (US$ ha^−1^) − cost of cultivation (US $ ha^−1^)]. Systems net returns were estimated by summing the net returns of both the crops (MCR rotation). The economics data (production costs/returns) were then converted from the Indian rupee (INR) to the US dollar (US$) based on the exchange rate during the respective years.

### System water productivity (SWP)

The SWP for the MCR across the years was computed by taking into account the total water input (irrigation + rainfall) during the growing seasons. The amount of rainfall water received was computed using the manual rain gauge data of meteorological observatories adjacent to the field. Irrigation depth was measured by using an ordinary scale meter that had mm and cm marks. In each plot, the depth of water was measured at ten selected spots immediately after the irrigation. Based on the rainfall pattern, three to six irrigations were applied to the maize at the critical growth stages, while for chickpea one to two irrigations per crop season was given at the late vegetative/pod development stages. The water productivity (kg ha^−1^ mm^−1^) was computed as per Eq. (10)^71^. The system water productivity (SWP) was worked out by adding the water productivity of both the crops

10 $$ {\text{Water productivity}} = {{{\text{grain yield }}\left( {{\text{kg ha}}^{{ - {1}}} } \right)} \mathord{\left/ {\vphantom {{{\text{grain yield }}\left( {{\text{kg ha}}^{{ - {1}}} } \right)} {{\text{total water applied }}\left( {{\text{mm}}} \right)}}} \right. \kern-\nulldelimiterspace} {{\text{total water applied }}\left( {{\text{mm}}} \right)}} $$

### Sustainable yield index (SYI)

Singh et al. and Wanjari et al.^72,73^ described the SYI as a quantitative measure of the sustainability of any agricultural system/practice. Using this concept, sustainability could be interpreted on the based on standard deviation (σ), where the lower values of the σ indicate the higher sustainability and vice-versa. Total crop productivity of maize and chickpea under different CEP and nutrient management was computed based on the 7 years' mean yield data. SYI was computed based on Eq. (11)^73^

11 $$ {\text{SYI}} = {{\left( {{-}\overline{{\text{y}}}_{{\text{a}}} {-}\sigma_{{{\text{n}}{-}{1}}} } \right)} \mathord{\left/ {\vphantom {{\left( {{-}\overline{{\text{y}}}_{{\text{a}}} {-}\sigma_{{{\text{n}}{-}{1}}} } \right)} {{\text{Y}}_{{\text{m}}}^{{{-}{1}}} }}} \right. \kern-\nulldelimiterspace} {{\text{Y}}_{{\text{m}}}^{{{-}{1}}} }} $$

where, $-\overline{{\text{y}}}_{{\text{a}}}$ is the average yield of crops across the years under specific management practice, σ_n−1_ is the standard deviation and Y_m_^−1^ is the maximum yield obtained under set a of practice.

### Statistical analyses

The significance of the treatment effects was determined through analysis of variance^74^. Pooled analysis was done for the grain/seed and stover yields after obtaining the significant differences in coefficient of variance of main and interaction effects over the years with the non-significant interaction effects between the years and the treatments. Turkey’s significant difference test was employed as a post hoc mean analysis at 5% level of significance using SAS 9.4 (SAS Institute, Cary, NC).

Authors have confirmed that all the plant studies were carried out in accordance with relevant national, international or institutional guidelines.

## Supplementary Information

Supplementary Table 1.
