# Supplementary material for: Long-term conservation agriculture and best nutrient management improves productivity and profitability coupled with soil properties of a maize–chickpea rotation
Source: Sci Rep. 2021 May 17;11:10386. doi: 10.1038/s41598-021-89737-9 (PMC8128923; doi:10.1038/s41598-021-89737-9)
Supplement: Supplementary file 1 — Supplementary Table 1. [file 41598_2021_89737_MOESM1_ESM.docx]

**Long-term conservation agriculture and best nutrient management improves productivity and profitability coupled with soil properties of a maize–chickpea rotation**

Vijay Pooniya^1*^, R.R. Zhiipao^1^, Niraj Biswakarma^1^, S.L. Jat^2^, Dinesh Kumar^1^, C.M. Parihar^1^, K. Swarnalakshmi^1^, Achal Lama^3^, A.K. Verma^1^, Debasish Roy^1^, Kajal Das^4^, K. Majumdar^5^, T. Satyanarayana^6^, R.D. Jat^7^, P.C. Ghasal^8^, Hardev Ram^9^, Rajkumar Jat^10^, Amlan Nath^1^

Supplementary table 1. Seven years monthly variations in max. (a) & min. (b) temperatures, and rainfall pattern (c) and relative humidity (d) during experimentation. ***Source:*** *Agromet. Observatory, Division of Agricultural Physics, IARI, New Delhi.*

(a)

| Month/year | July | Aug. | Sep. | Oct. | Nov. | Dec. | Jan. | Feb. | Mar. | Apr. |
| --- | --- | --- | --- | --- | --- | --- | --- | --- | --- | --- |
| 2013–14 | 34.8 | 32.8 | 34.5 | 31.5 | 26.9 | 22.4 | 18.6 | 21.0 | 26.9 | 34.8 |
| 2014–15 | 35.9 | 35.8 | 34.1 | 33.2 | 28.3 | 20.6 | 16.9 | 24.6 | 27.2 | 33.9 |
| 2015–16 | 33.8 | 33.5 | 35.2 | 33.8 | 28.1 | 22.6 | 20.7 | 24.6 | 30.8 | 38.7 |
| 2016–17 | 33.8 | 32.6 | 34.3 | 33.9 | 29.0 | 23.3 | 20.1 | 23.9 | 29.8 | 38.0 |
| 2017–18 | 34.1 | 33.6 | 33.6 | 34.0 | 26.8 | 23.0 | 20.6 | 24.9 | 31.6 | 36.5 |
| 2018–19 | 35.7 | 33.6 | 34.0 | 30.2 | 27.4 | 19.4 | 20.0 | 21.1 | 27.0 | 37.2 |
| 2019–20 | 41.3 | 38.2 | 34.0 | 35.1 | 32.0 | 27.0 | 15.2 | 20.0 | 21.0 | 35.5 |

(b)

| Month/year | July | Aug. | Sep. | Oct. | Nov. | Dec. | Jan. | Feb. | Mar. | Apr. |
| --- | --- | --- | --- | --- | --- | --- | --- | --- | --- | --- |
| 2013–14 | 25.7 | 25.1 | 24.0 | 19.2 | 9.9 | 7.1 | 6.8 | 7.5 | 12.7 | 17.9 |
| 2014–15 | 26.3 | 25.8 | 24.0 | 18.8 | 10.6 | 6.7 | 6.8 | 10.6 | 13.1 | 19.2 |
| 2015–16 | 25.7 | 25.5 | 23.6 | 17.5 | 11.9 | 6.1 | 6.5 | 8.1 | 13.7 | 19.1 |
| 2016–17 | 24.4 | 24.2 | 22.5 | 16.2 | 9.0 | 5.3 | 7.7 | 9.9 | 14.2 | 20.7 |
| 2017–18 | 26.2 | 25.9 | 23.7 | 17.2 | 10.6 | 6.8 | 4.3 | 8.4 | 13.4 | 19.9 |
| 2018–19 | 27.5 | 26.5 | 25.5 | 14.8 | 13.8 | 7.5 | 6.4 | 9.6 | 12.4 | 21.1 |
| 2019–20 | 29.0 | 28.2 | 26.1 | 24.0 | 18.5 | 14.0 | 6.0 | 6.0 | 8.0 | 20.2 |

©

| Month/Year | July | Aug. | Sep. | Oct. | Nov. | Dec. | Jan. | Feb. | Mar. | Apr. |
| --- | --- | --- | --- | --- | --- | --- | --- | --- | --- | --- |
| 2013–14 | 459.8 | 521.9 | 108.1 | 109.0 | 0.4 | 6.8 | 18.6 | 63.5 | 63.5 | 16.4 |
| 2014–15 | 227.8 | 98.9 | 124.3 | 0.0 | 0.0 | 26.4 | 35.8 | 0.0 | 201.8 | 51.8 |
| 2015–16 | 377.8 | 261.3 | 67.2 | 0.0 | 2.2 | 0.0 | 0.0 | 1.4 | 17.8 | 0.6 |
| 2016–17 | 540.9 | 388.2 | 173.4 | 37.8 | 0.0 | 0.0 | 64.8 | 0.0 | 17.1 | 8.0 |
| 2017–18 | 170.2 | 202.0 | 193.0 | 0.0 | 0.0 | 7.4 | 6.0 | 0.0 | 0.0 | 26.0 |
| 2018–19 | 368.4 | 247.7 | 237.9 | 0.0 | 4.0 | 0.0 | 52.0 | 70.8 | 10.2 | 5.7 |
| 2019–20 | 283.9 | 277.0 | 17.4 | 41.0 | 7.4 | 66.0 | 47.7 | 2.0 | 174.6 | 8.8 |

(d)

| Month/year | July | Aug. | Sep. | Oct. | Nov. | Dec. | Jan. | Feb. | Mar. | Apr. |
| --- | --- | --- | --- | --- | --- | --- | --- | --- | --- | --- |
| 2013–14 | 81.0 | 89.0 | 75.0 | 75.0 | 70.0 | 75.0 | 82.0 | 80.0 | 69.0 | 57.0 |
| 2014–15 | 75.0 | 72.0 | 71.0 | 62.0 | 61.0 | 76.0 | 83.0 | 70.0 | 71.0 | 60.0 |
| 2015–16 | 76.0 | 78.0 | 63.0 | 64.0 | 69.0 | 72.0 | 78.0 | 71.0 | 71.0 | 56.0 |
| 2016–17 | 82.0 | 82.0 | 72.0 | 63.0 | 63.0 | 72.0 | 76.4 | 70.0 | 62.0 | 55.7 |
| 2017–18 | 78.2 | 82.4 | 76.8 | 67.8 | 69.4 | 68.9 | 71.7 | 66.3 | 57.5 | 45.1 |
| 2018–19 | 83.3 | 89.0 | 87.3 | 89.0 | 88.6 | 86.7 | 90.3 | 94.7 | 89.3 | 71.8 |
| 2019–20 | 65.0 | 84.0 | 90.2 | 86.0 | 87.0 | 87.0 | 67.2 | 93.5 | 98.0 | 65.5 |
